# Supplementary material for: Getting More Out of Biomedical Documents with GATE's Full Lifecycle Open Source Text Analytics
Source: PLoS Comput Biol. 2013 Feb 7;9(2):e1002854. doi: 10.1371/journal.pcbi.1002854 (PMC3567135; doi:10.1371/journal.pcbi.1002854)
Supplement: Dataset S1 — GATE software. Dataset S1 bundled with this paper contains a distribution of GATE (or see http://gate.ac.uk/download/). (TGZ) [file pcbi.1002854.s001.tgz › plos-gate/doc/announce-5-0.html]

GATE Version 5.0 release (May 2009)


# GATE Version 5.0 release (May 2009)

# 1. Major new features

## 1.1. JAPE language improvements

GATE 5.0 introduces several new extensions to the JAPE language to make it more
flexible:

- "Negative" constraints, to
  support patterns like "match Person annotations, but only where they do
  not start with an upper-case initial word".
- A richer set of matching
  operators for feature values, e.g. {Token.length < 5}.
- "Meta-property" accessors, giving access to things like the document text
  covered by an annotation, e.g. {Organization@string == "IBM"}.
- Contextual
  operators, allowing you to search for one annotation contained within (or
  containing) another.
- Bounded range Kleene operators, e.g. ({Token})[2,5] to match between 2 and
  5 consecutive tokens.
- Additional operators can be added via runtime configuration.

Montreal transducer users will be familiar with some of these operators, this
release brings support for them into the core of GATE.

## 1.2. Resource configuration via Java 5 Annotations

This release introduces the ability to configure resource classes via
source-level
annotations, to complement (but not replace) the creole.xml mechanism.

## 1.3. Ontology-based gazetteer

A new plugin which
matches terms taken from an OWL ontology. In combination with a few other
generic GATE resources this can produce ontology-aware annotations over text.

## 1.4. Tools for inter-annotator agreement and merging

Three new plugins to support manual annotation tasks with several annotators
working on the same documents. The plugins can
copy annotations
from several parallel documents into one master document, compute
inter-annotator agreement
scores between the different annotators, and
merge the annotations
into a single "consensus" set.

## 1.5. Packaging applications for GATE Teamware

A menu item and underlying
Ant task to assemble a saved
GATE application along with all the resource files it uses into a single
self-contained package to run on another machine (for example as a
GATE Teamware service).

# 2. GUI improvements

Many elements of the GATE GUI (now called GATE-Developer) have been overhauled
to be more user-friendly, with context-sensitive help, a "search and annotate"
function in the document editor, drag and drop support in appropriate places,
etc. In addition, there is a new
schema-driven
tool to streamline manual annotation tasks.

# 3. Other new features and improvements

Many smaller features have also been added. The full list is in the
changelog, and includes the
following:

- Real-time corpus controller,
  which terminates processing of a document if it takes longer than a
  configurable timeout, useful for large batch processes.
- A new sentence splitter
  based on regular expressions.
- Re-designed ANNIC GUI.

...and many more.

Full details of this release can be found in the
changelog. To download GATE,
visit the download page.
